# Supplementary material for: Analysis of Primary Liquid Chromatography Mass Spectrometry Data by Neural Networks for Plant Samples Classification
Source: Metabolites. 2022 Oct 19;12(10):993. doi: 10.3390/metabo12100993 (PMC9611620; doi:10.3390/metabo12100993)

Analysis of Primary Liquid Chromatography Mass Spectrometry Data by Neural Networks for Plant Samples Classification

Polina Turova [1,*], Andrey Stavrianidi [1,2], Viktor Svekolkin [3], Dmitry Lyskov [4], Ilya Podolskiy [5], Igor Rodin [1], Oleg Shpigun [1,2] and Aleksey Buryak [2]

[1] Faculty of Chemistry, M.V. Lomonosov Moscow State University, 1-3 Leninskie Gory, Moscow 119991, Russia
[2] A.N. Frumkin Institute of Physical Chemistry and Electrochemistry, Russian Academy of Sciences, 31-4 Leninsky Prospect, Moscow 119071, Russia
[3] BostonGene Corporation, University Office Park III, 95 Sawyer Road, Waltham, MA 02453, USA
[4] Faculty of Biology, M.V. Lomonosov Moscow State University, 1-12 Leninskie Gory, Moscow 119234, Russia
[5] Bruker Ltd., Pyatnitskaya 50/2 Build. 1, Moscow 119017, Russia
* Correspondence: turova.polina@gmail.com

Figure S1. Overlay of mass chromatograms of (A) roots samples containing **compound 1** (8.96 min, m/z 307); (B) all other samples which are not containing **compound 1**; (C) stems samples containing **compound 3** (10.37 min, m/z 607); (D) all other samples which are not containing **compound 3**.

Figure S2. MS spectrum of **compound 1**

Figure S3. MS/MS spectrum of **compound 1** precursor ion, m/z 599

Figure S4. MS/MS spectrum of **compound 1** precursor ion, m/z 305

Figure S5. MS spectrum of **compound 2**

Figure S6. MS/MS spectrum of **compound 2** precursor ion, m/z 325

Figure S7. MS spectrum of **compound 3**

Figure S8. MS/MS spectrum of **compound 3** precursor ion, m/z 607

Figure S9. MS spectrum of **compound 4**

Figure S10. MS/MS spectrum of **compound 4** precursor ion, m/z 603

Figure S11. MS spectrum of **compound 5**

Figure S12. MS/MS spectrum of **compound 5** precursor ion, m/z 625

Figure S13. MS spectrum of **compound 6**

Figure S14. MS/MS spectrum of **compound 6** precursor ion, m/z 621

Figure S15. MS spectrum of **compound 7**

Figure S16. MS/MS spectrum of **compound 7** precursor ion, m/z 522

Figure S17. MS spectrum of **compound 8**

Figure S18. MS/MS spectrum of **compound 8** precursor ion, m/z 520

Figure S19. TIC chromatograms of roots sample

Figure S20. TIC chromatograms of stems sample

Figure S21. TIC chromatograms of leaves sample

Figure S22. TIC chromatograms of fruits sample

Figure S1. Overlay of mass chromatograms of (A) roots samples containing **compound 1** (8.96 min, m/z 307); (B) all other samples which are not containing **compound 1**; (C) stems samples containing **compound 3** (10.37 min, m/z 607); (D) all other samples which are not containing **compound 3**.

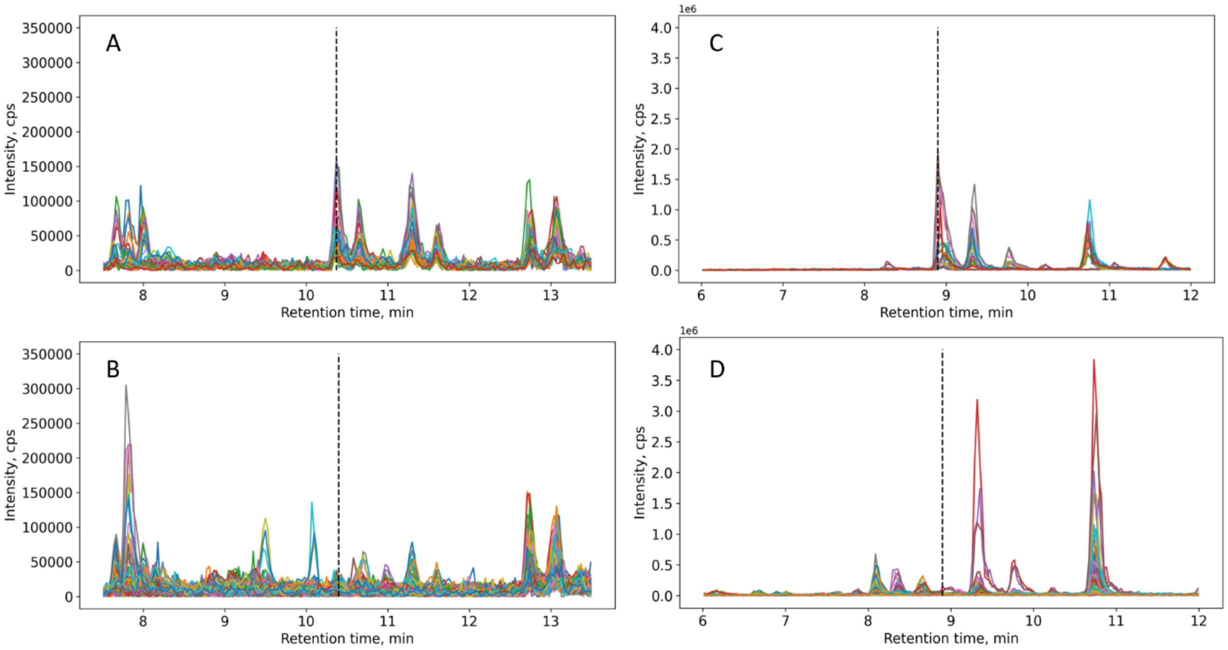

Figure S2. MS spectrum of **compound 1**

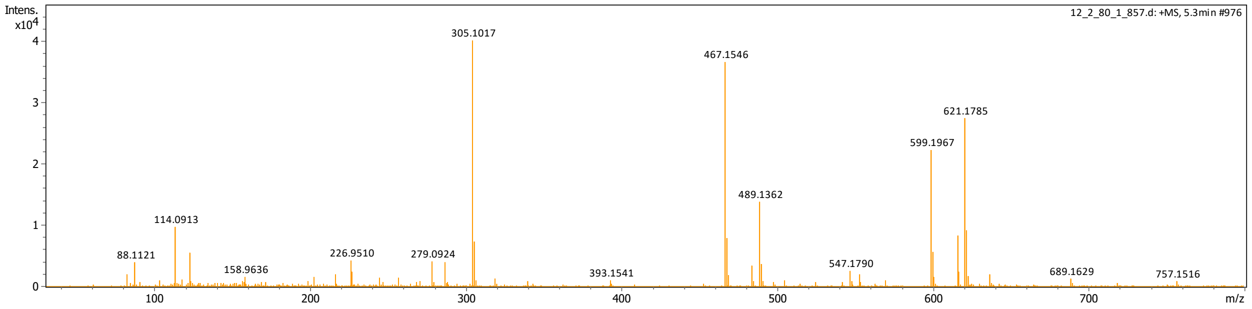

Figure S3. MS/MS spectrum of **compound 1** precursor ion, m/z 599

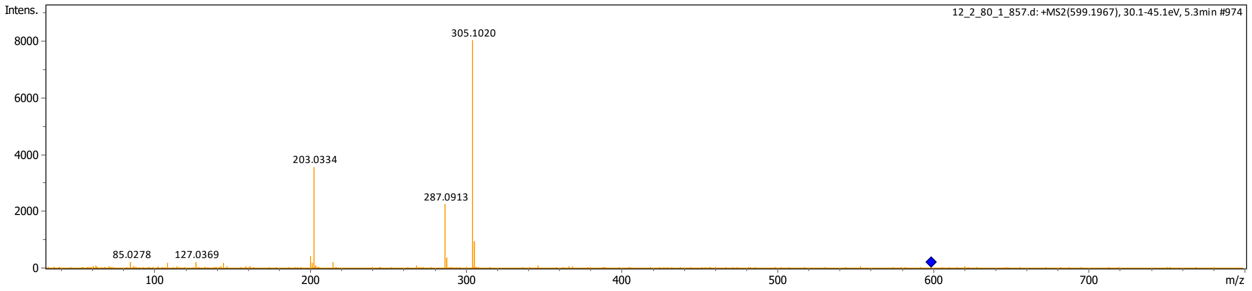

Figure S4. MS/MS spectrum of **compound 1** precursor ion, m/z 305

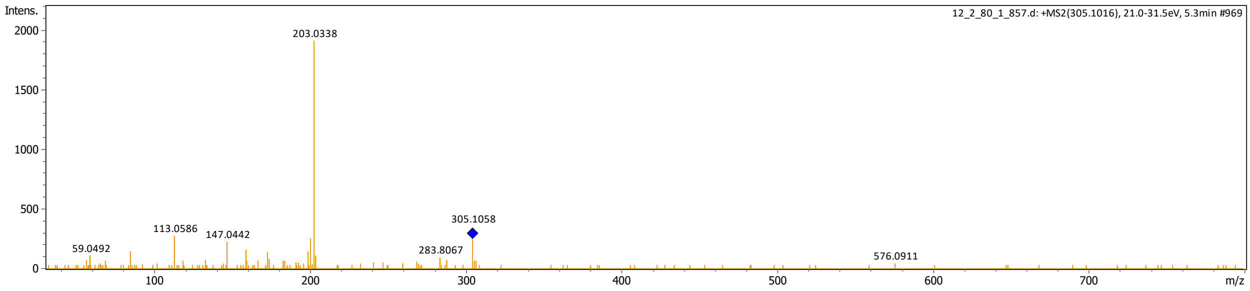

Figure S5. MS spectrum of **compound 2**

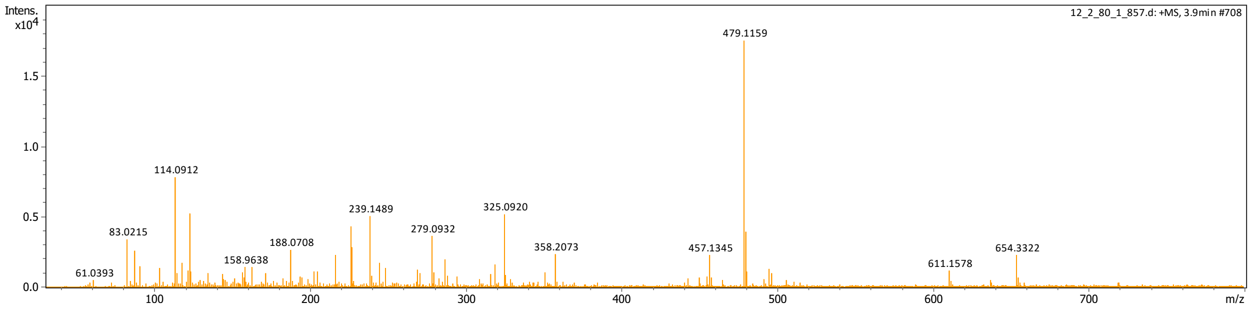

Figure S6. MS/MS spectrum of **compound 2** precursor ion, m/z 325

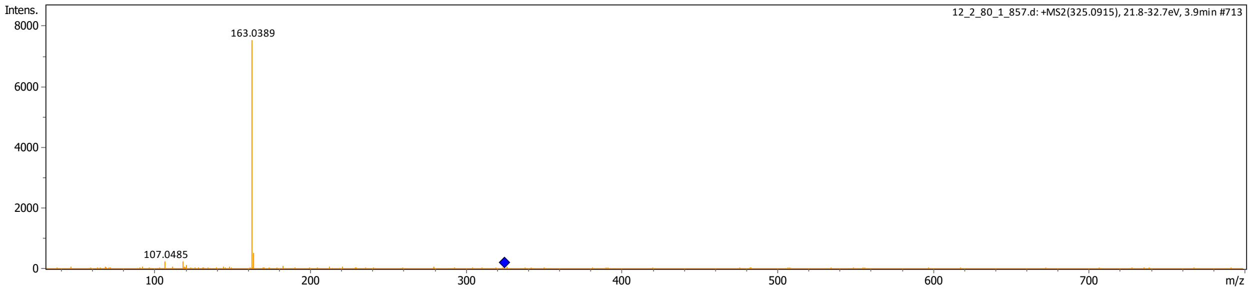

Figure S7. MS spectrum of **compound 3**

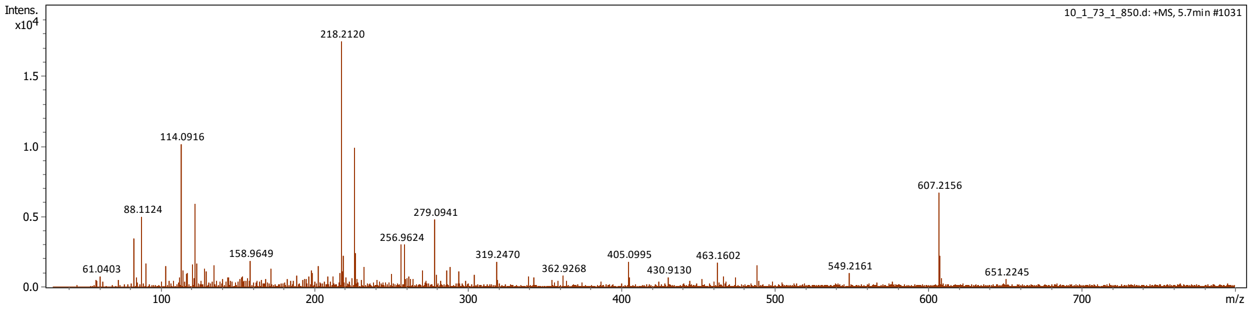

Figure S8. MS/MS spectrum of **compound 3** precursor ion, m/z 607

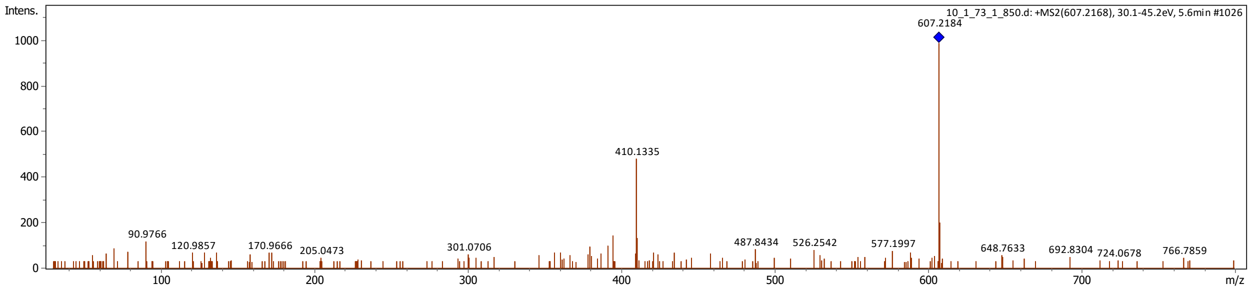

Figure S9. MS spectrum of **compound 4**

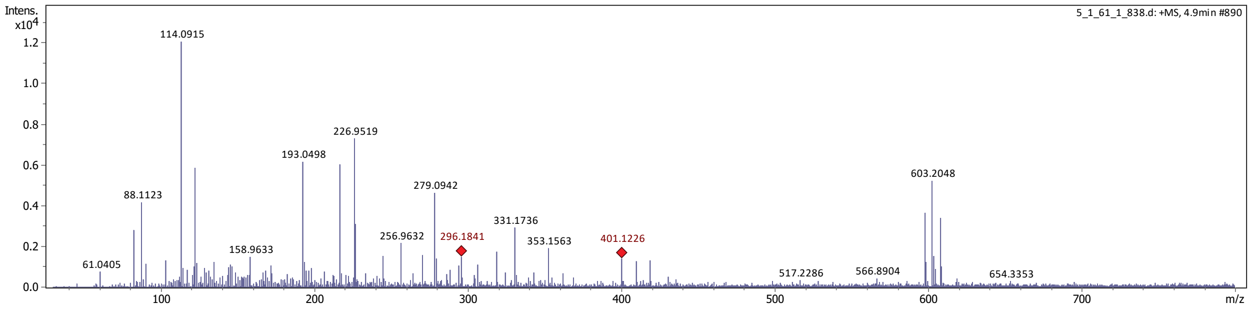

Figure S10. MS/MS spectrum of **compound 4** precursor ion, m/z 603

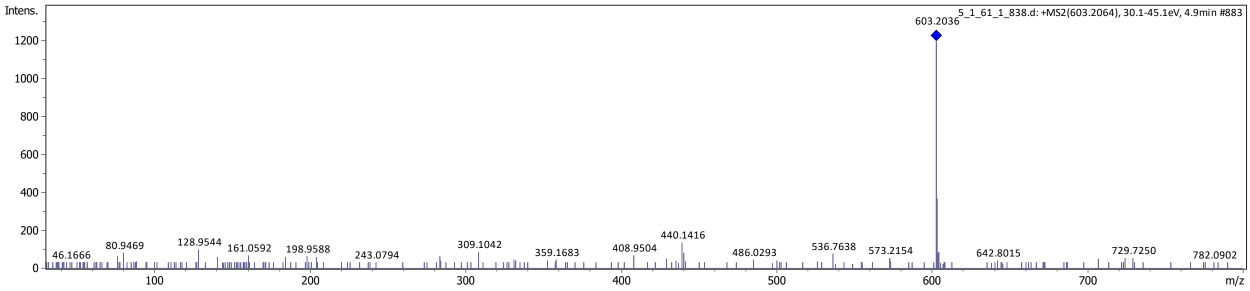

Figure S11. MS spectrum of **compound 5**

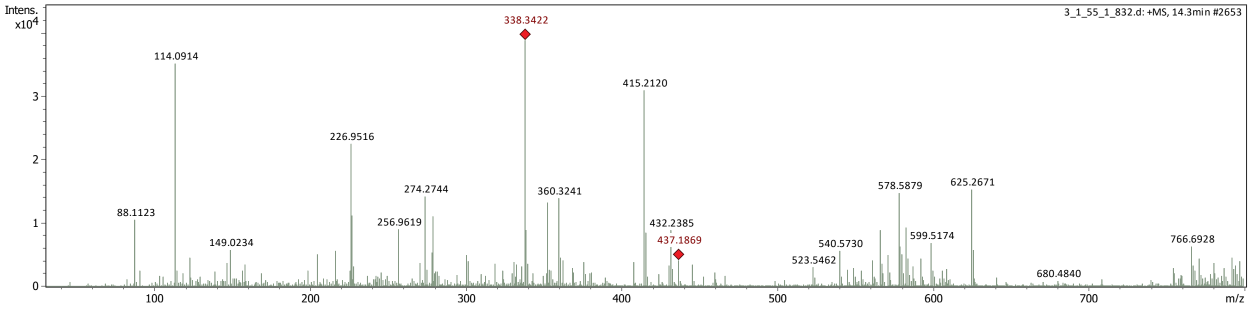

Figure S12. MS/MS spectrum of **compound 5** precursor ion, m/z 625

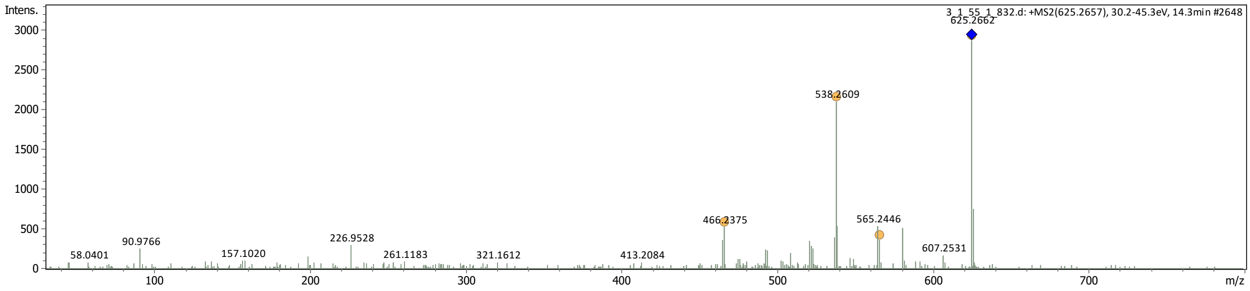

Figure S13. MS spectrum of **compound 6**

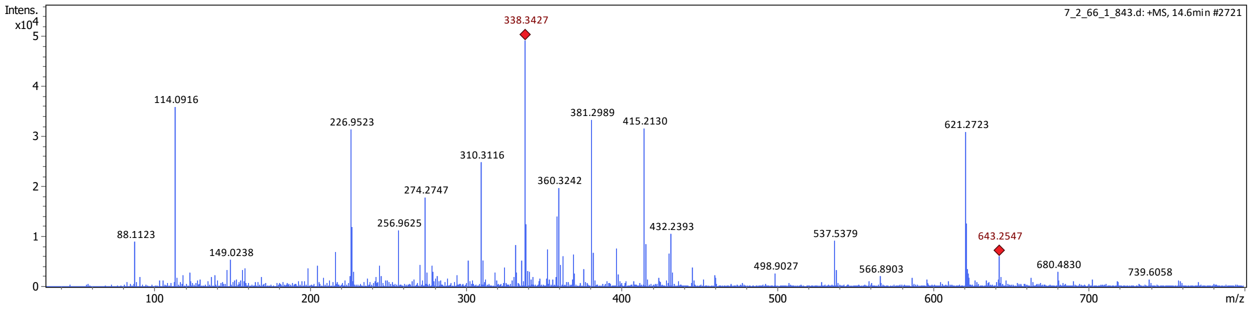

Figure S14. MS/MS spectrum of **compound 6** precursor ion, m/z 621

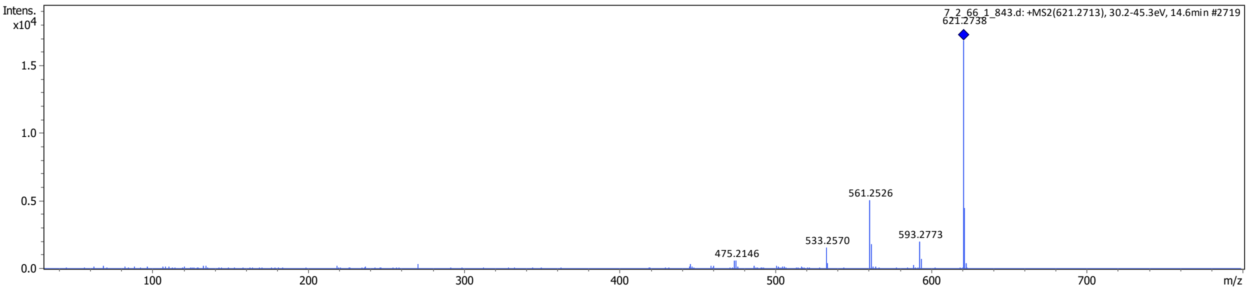

Figure S15. MS spectrum of **compound 7**

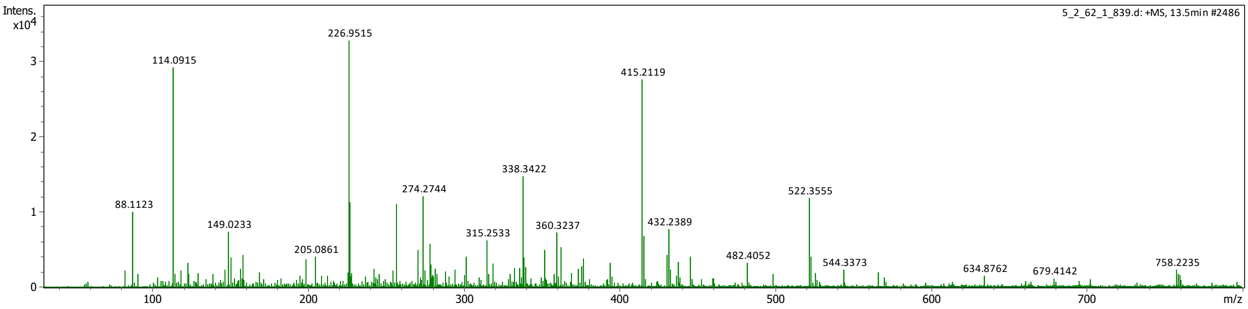

Figure S16. MS/MS spectrum of **compound 7** precursor ion, m/z 522

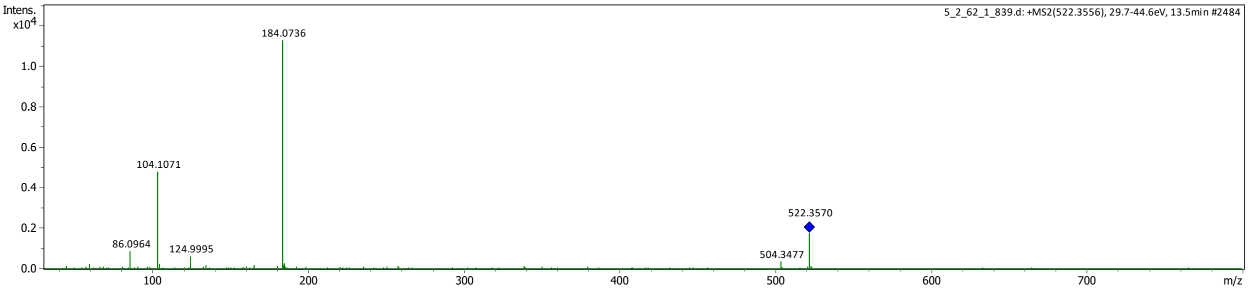

Figure S17. MS spectrum of **compound 8**

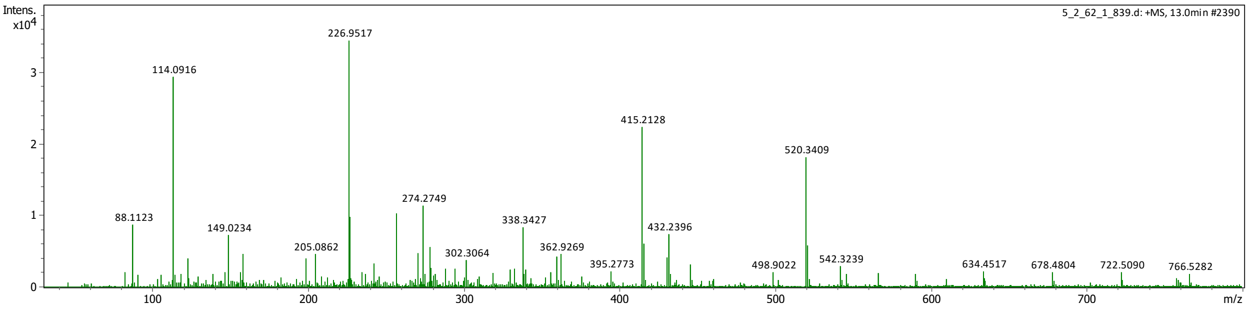

Figure S18. MS/MS spectrum of **compound 8** precursor ion, m/z 520

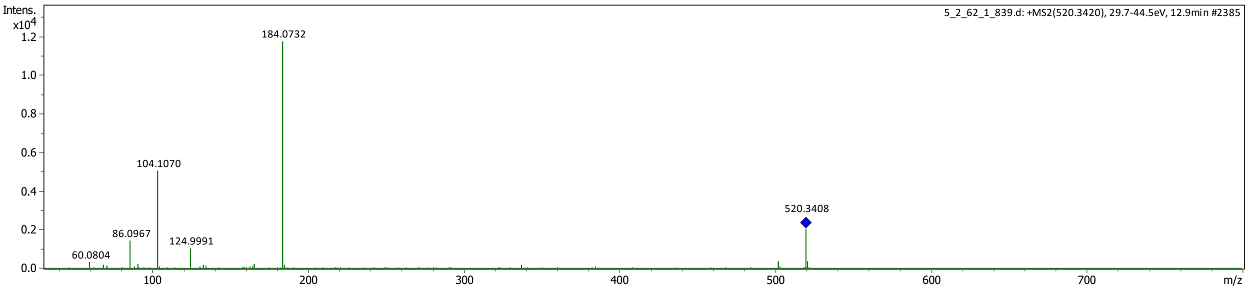

Figure S19. TIC chromatograms of roots samples

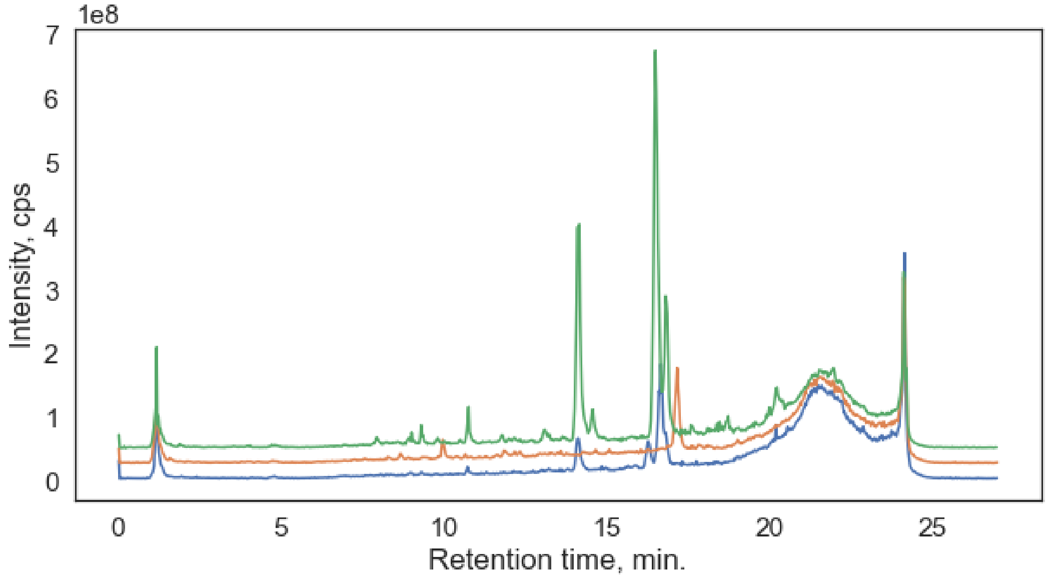

Figure S20. TIC chromatograms of stems samples

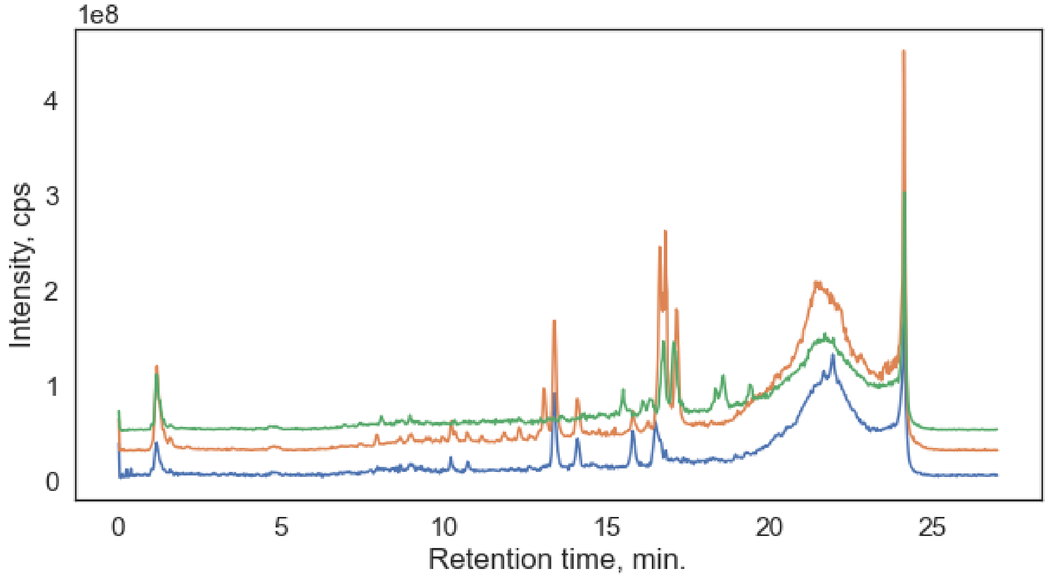

Figure S21. TIC chromatograms of leaves samples

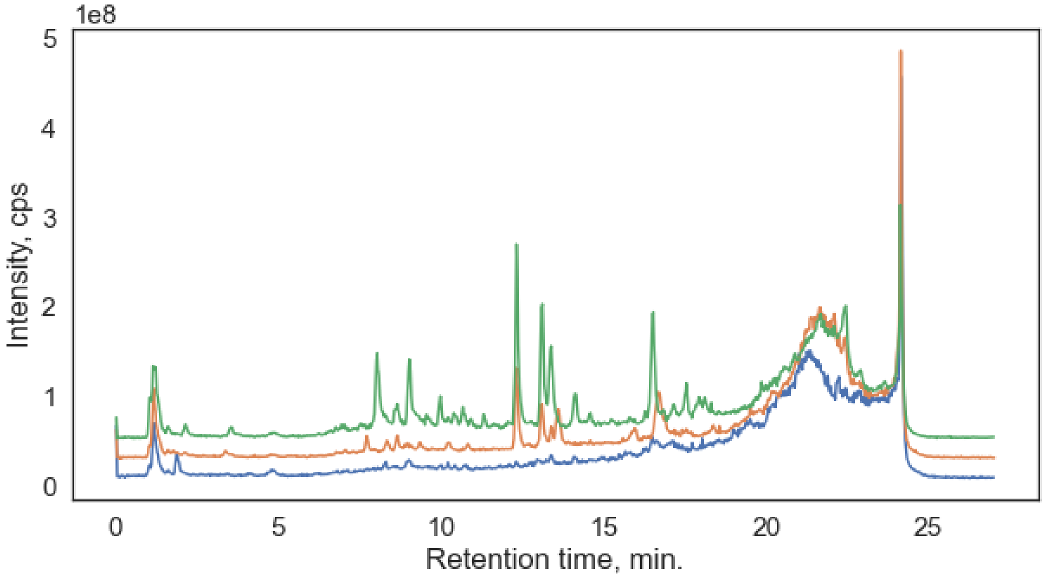

Figure S22. TIC chromatograms of fruits samples

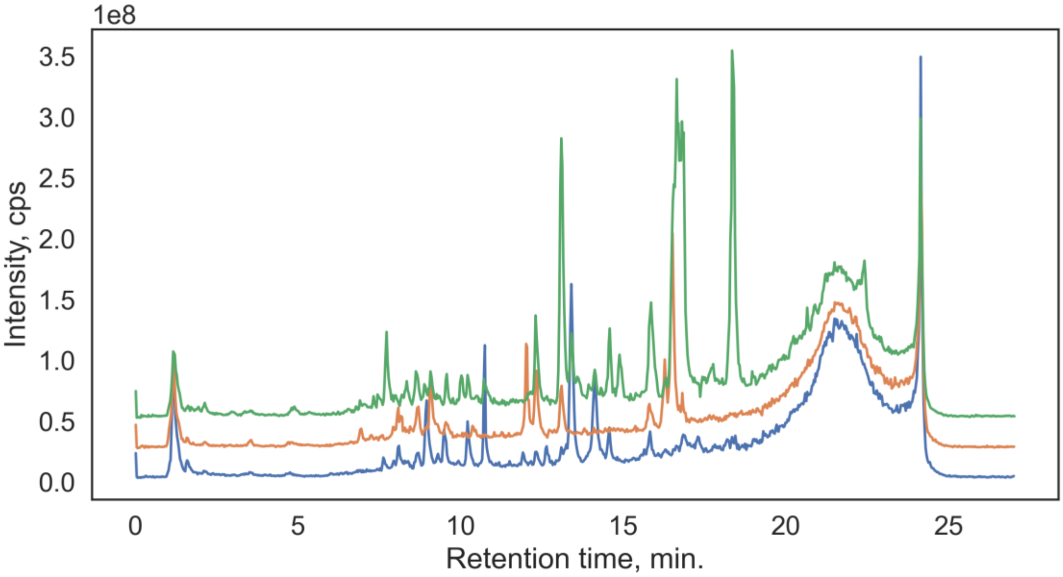

Supplement: Supplementary file 1 [file metabolites-12-00993-s001.zip › metabolites-1945014-supplementary.pdf]
